# Downregulation of lncRNA APCDD1L-AS1 due to DNA hypermethylation and loss of VHL protein expression promotes the progression of clear cell renal cell carcinoma

**DOI:** 10.7150/ijbs.71519

**Published:** 2022-03-21

**Authors:** Wuping Yang, Jingcheng Zhou, Zedan Zhang, Kenan Zhang, Yawei Xu, Lei Li, Lin Cai, Yanqing Gong, Kan Gong

**Affiliations:** 1 Department of Urology, Peking University First Hospital, Beijing 100034, P.R. China.; 2 Hereditary Kidney Cancer Research Center, Peking University First Hospital, Beijing 100034, P.R. China.; 3 Institute of Urology, Peking University, Beijing 100034, P.R. China.

**Keywords:** long non-coding RNA, APCDD1L-AS1, clear cell renal cell carcinoma, DNA hypermethylation, von Hippel Lindau

## Abstract

**Background:** The current studies only indicated that long non-coding RNA (lncRNA) APCDD1L-AS1, as a novel lncRNA, may play a role in oral squamous cell carcinoma and lung cancer. However, its potential role in clear cell renal cell carcinoma (ccRCC) and its possible mechanism of action remain vague.

**Methods:** TCGA-KIRC and GEO data and qRT-PCR and pyrosequencing results of clinical specimens were used to identify the expression level and DNA methylation status of APCDD1L-AS1. The effects of APCDD1L-AS1 overexpression on ccRCC growth and metastasis were determined by function experiments. Western blot and Tandem mass tags (TMT) were utilized to explore the relationship between APCDD1L-AS1 and VHL expression and its downstream underlying mechanisms.

**Results:** The expression of APCDD1L-AS1 was downregulated in ccRCC. Decreased APCDD1L-AS1 expression was related to higher tumor stage and histological grade and shorter RFS (Relapse-free survival). Besides, APCDD1L-AS1 overexpression restrained the growth and metastasis of ccRCC cells *in vitro and in vivo*. Moreover, reduced APCDD1L-AS1 expression could be caused by DNA hypermethylation and loss of von Hippel Lindau (VHL) protein expression. Furthermore, the dysregulation of histones expression caused by APCDD1L-AS1 overexpression may be one of the important mechanisms to suppress the progression of ccRCC.

**Conclusion:** APCDD1L-AS1 was able to inhibit the progression of ccRCC, and its decreased expression could be caused by DNA hypermethylation and loss of VHL protein expression. Therefore, APCDD1L-AS1 may serve as a new therapeutic target in the treatment of ccRCC.

## Introduction

Renal cell carcinoma (RCC), one of the three major tumors of the urinary system, accounts for 2-3% of global cancer diagnoses and deaths, and its incidence has been on the rise for over 20 years 1. Besides, 60% of patients are diagnosed at the metastatic stage or develop metastases during the disease process 2, 3, and the 5-year survival rate for patients with metastatic RCC is as low as 12% 4.

There are several subtypes of RCC, and more than 70% of individuals are diagnosed with clear cell RCC (ccRCC) 5. Loss or mutation of the von Hippel Lindau (*VHL*) gene is generally regarded as one of the inevitable initial steps in the development of ccRCC. VHL protein is an E3 ubiquitin ligase, and its best-known function is the ubiquitination of the prolyl hydroxylation transcription factors hypoxia-inducible factors (HIF1α and HIF2α) 6, 7, and leads to their subsequent proteolytic degradation under normoxic conditions 8. HIF1α and HIF2α can regulate the transcription of many genes involved in angiogenesis, metabolism and chromatin remodeling, which are associated with the development of ccRCC 9. Based on this biological property of ccRCC, several targeted drugs with antiangiogenic activity have been approved for the treatment of advanced RCC, including mammalian target of rapamycin inhibitors, monoclonal antibody that interferes with vascular endothelial growth factor and tyrosine kinase inhibitors 10. Unfortunately, current drug treatments are not effective enough, have severe side effects, and are prone to developing drug resistance during the course of treatment. Therefore, there is an urgent need to find more effective and safe therapeutic targets for ccRCC.

In recent years, numerous studies have proved that long non-coding RNA (lncRNA) plays a crucial role in the carcinogenesis and protein coding gene expression disorders of multiple tumors, and they can act as both oncogenes and tumor suppressors 11. Currently, lncRNAs are known to be involved in various cellular processes including cell differentiation, autophagy, apoptosis, chemoresistance and metastasis 12-14. Moreover, lncRNAs also play important roles in the development of ccRCC. For example, ZNF582-AS1 restrained the growth and metastasis of ccRCC by regulating the N(6)-methyladenosine modification of MT-RNR1 15, and PVT1 promoted the tumorigenesis and metastasis of ccRCC by stabilizing HIF2α 16. APCDD1L-AS1 was a novel lncRNA and located at Chromosome 20: 58,515,379-58,619,888. Previous studies have shown that APCDD1L-AS1 was related to the prognosis of patients with lung squamous cell carcinoma, induced 5-fluorouracil resistance in oral squamous cell carcinoma (OSCC) and icotinib resistance in lung adenocarcinoma (LUAD) 17-19. Nevertheless, the explicit role of APCDD1L-AS1 in ccRCC is still vague.

In the present study, based on data from TCGA-KIRC and GEO databases and qRT-PCR results of clinical tumor specimens, our results suggested that the expression of lncRNA APCDD1L-AS1 was significantly lower in ccRCC than that in adjacent normal renal (AN) tissue. Overexpression of APCDD1L-AS1 increased cell apoptosis and inhibited cell proliferative, migratory and invasive ability *in vitro* and* in vivo*. Moreover, reduced expression of APCDD1L-AS1 could be caused by DNA hypermethylation and loss of VHL protein expression in ccRCC. Furthermore, APCDD1L-AS1 overexpression could lead to alterations in the expression of histones.

## Materials and methods

### Ethics statement

This study was approved by the ethics committee of Ministry of Science and Technology of the People's Republic of China (Approval no. 2021SLCJ2189, 2021.09.14, Beijing, China), and conducted in accordance with the Declaration of Helsinki (1975). Informed consent signed by each patient has been obtained in this study.

### TCGA-KIRC and GEO data acquisition

Transcriptome sequencing data and DNA methylation data of ccRCC patients were gained from TCGA-KIRC (The Cancer Genome Atlas-Kidney Renal Clear Cell Carcinoma). GSE53757, GSE66272 and GSE105260 data were obtained from Gene Expression Omnibus (GEO) DataSets.

### Clinical samples collection

Samples from 54 patients diagnosed with ccRCC were collected in the study. All samples were provided by the Department of Urology, Peking University First Hospital. Clinicopathological and survival information data of these 54 ccRCC cases were also obtained.

### Cell culture

The normal human renal tubular epithelial cell line HK2, and six RCC cell lines A498, ACHN, OSRC2, Caki-1, 786-O and RCC4 were used in this study, and these cell lines were cultured according to conditions specified by the provider. APCDD1L-AS1 overexpression plasmid, VHL overexpression and knockdown plasmids, HIF1α and HIF2α knockdown plasmids and HIF1α overexpression plasmid were constructed. Accordingly, the stably transfected cell lines were established by lentivirus infection.

### Quantitative real-time PCR (qRT-PCR)

Total RNA of 54 pairs of ccRCC tissue specimens and the transfected cell lines was extracted using the TRIzol reagent (Invitrogen; USA). cDNA was produced using the RevertAid First Strand cDNA Synthesis Kit (Thermo, K1622, USA). qRT-PCR was performed according to the manufacturer's recommended procedure, and normalized to β-actin. All experiments were repeated at least three times. The primer sequences of APCDD1L-AS1 are as follows: Forward primer: GTTCCTGCTCGGTTTCTGGA; Reverse primer: TTTTGCCTGCACAGCATTCC.

### Cell proliferation assays

EdU Apollo DNA *in vitro* kit (RiboBio, Guangzhou, China) and BeyoClick™ EdU Cell Proliferation Kit with DAB (Beyotime, China) were utilized to identify the cell proliferation ability. Besides, the proliferation ability of cells was also examined using the MTT Cell Proliferation and Cytotoxicity Assay Kit (Beyotime, China).

### Cell apoptosis assays

One Step TUNEL Apoptosis Assay Kit (Beyotime, China) was utilized to test cell apoptotic status in cell slides. Colorimetric TUNEL Apoptosis Assay Kit (Beyotime, China) was utilized to examine cell apoptotic status in the paraffin sections of mice tumors. In addition, cell apoptosis was also assayed by staining with Annexin V-FITC and PI (Beyotime, China), and a flow cytometer was utilized to detect the fluorescence level of the cells.

### Cell cycle assays

Cell cycle was detected using the Cell Cycle and Apoptosis Analysis Kit (Beyotime, China) according to the instructions.

### Cell transwell migratory and invasive assays

For cell transwell migration assay, 2×10^3^ OSRC2 and Caki-1 cells were plated into the upper chambers with 150 μL serum-free DMEM. The lower chambers were filled with 650 μL DMEM containing 15% FBS. After 48 h, cells under the surface of the lower chamber were washed with PBS and stained with 0.5% crystal violet for 20 min. For cell invasion assay, 2×10^3^ cells were seeded on upper chambers coated with 150 μL Matrigel (1:6 dilution in PBS). The culture conditions were the same as transwell migration assay. 48 h later, adherent cells on the lower surface were stained with 0.5% crystal violet for 20 min. The number of cells on the lower surface was then counted under a microscope.

### Wound healing assay

Cell migration ability was also determined by wound-healing assay. Briefly, approximately 1×10^6^ cells were seeded in 6-well plates at equal densities and grown to 85% ~ 95% confluency. Then, artificial gaps were generated by a 1 ml sterile pipette tip after transfection with the corresponding lentivirus. Wounded areas were marked and photographed under a microscope.

### Mouse model experiments

Eighteen 4-week-old male BALB/c nude mice were purchased from Vitalriver, Beijing, China. About 5×10^6^ APCDD1L-AS1 overexpressed OSRC2 cells and its control cells were implanted subcutaneously into the right side of the mice. Tumor size was measured every four days and calculated using the formula: (length×width^2^)/2. For EdU incorporation assay, ethynyl-2-deoxyuridine (EdU, 50 mg/kg; Beyotime, China) was intraperitoneally injected three hours before mice were euthanized. For the lung metastasis experiment, ten 5-week-old male B-NDG severely immunodeficient mice were purchased from BIOCYTOGEN, Beijing, China. About 50×10^5^ CON-Luc and APCDD1L-AS1-Luc Caki-1 cells were suspended in 100 ul PBS and injected into the lateral tail veins of each mouse. Forty days after injection, mice were anesthetized with tribromoethanol, and bioluminescence imaging was performed as described previously 15. The procedures of animal experiments were approved by the Institutional Animal Care and Use Committee at Peking University First Hospital following the Guideline for the Care and Use of Laboratory Animals (Approval no. 202056, 2020.09.01, Beijing, China).

### Immunohistochemistry

Immunohistochemical staining was utilized to measure the protein expression levels of E-cadherin and N-cadherin in the paraffin sections of mice lung metastases, and the detailed primary antibody information is as follows: anti-E-cadherin (1:500; Abcam, ab40772) and anti-N-cadherin (1:1000; Abcam, ab19348).

### Pyrosequencing

Pyrosequencing was performed as described previously 20. The primer sequences of used for pyrosequencing are as follows: F1: GTTTAATTTGTTAGAAGAGGTGGAGATA; R1: CCAAAACTTAAAAAAAACCTCCTTAC; S1: AAAACCTCCTTACCCA.

### Western blot

RIPA lysis buffer was used to extract the total protein form cells and tissues, and the protein level was quantified using the BCA protein assay Kit (APPLYGEN). The detailed information on the primary antibodies used is as follows: anti-Cleaved Caspase-3 (1:1000, 9664S, CST), anti-Bcl-2 (1:2000, Proteintech, China), anti-VHL (1:1000, 68547S, CST); anti-HIF1α (1:1000, 36169S, CST), anti-HIF2α (1:1000, 71565S, CST); anti-Histone H3.1 (1:1000, NBP2-75524, Novus Biologicals), Histone H4 (1:1000, Proteintech, China), Histone H3 (1:1000, Proteintech, China), Histone H1.3 (1:1000, ab183736, Abcam), Histone H1.5 (1:1000, DF13539, Affinity), Histone H1.4 (1:1000, A20550, ABclonal), Histone H1.2 (1:1000, Proteintech, China), and anti-GAPDH (1:8000, Proteintech, China).

### TMT (Tandem mass tags) quantitative proteomics test

The TMT test was completed by the Beijing Liuhe BGI Technology Co., LTD. The brief steps were as follows: (1) Pre-experimental stage: sample protein extraction, protein quantification and gel electrophoresis, FASP enzymatic hydrolysis, pre-mass spectrometry analysis, database comparison, and pre-experiment pass. (2) Formal experimental stage: peptide segment TMT labeling, High PH RP classification, mass spectrometry analysis, database comparison, peptide segment mass deviation, bioinformatics analysis and formal experiment report.

### Statistical analyses

Student's t-test was used to detect differences in continuous variables. Pearson's correlation analysis was utilized to examine the correlation between APCDD1L-AS1 expression and VHL expression, the DNA methylation level of cg23487201 and APCDD1L-AS1 expression. All statistical tests were two-sided, and a *P* value of < 0.05 was regarded as statistical difference.

## Results

### The expression of lncRNA APCDD1L-AS1 in ccRCC was significantly downregulated

**Figure 1:**
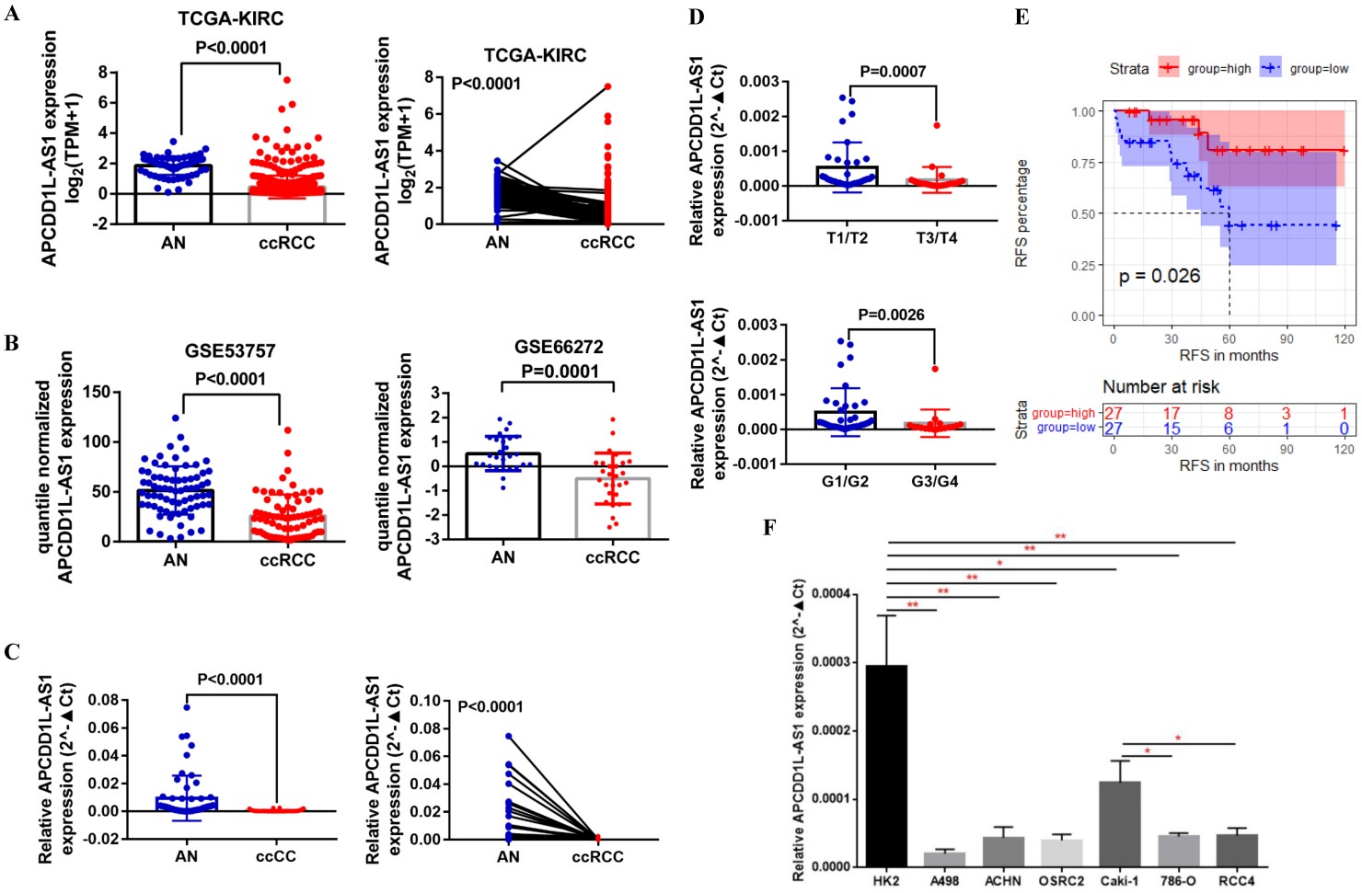
** APCDD1L-AS1 expression was decreased in ccRCC. A** Comparison of APCDD1L-AS1 expression in ccRCC (n=539) and adjacent normal renal (AN) tissues (n=72) based on TCGA-KIRC data. **B** Comparison of APCDD1L-AS1 expression in ccRCC and AN tissue based on GSE53757 (n=72) and GSE66272 (n=27) data. **C and D** Comparison of APCDD1L-AS1 expression in 54 pairs of ccRCC and AN tissue, and its correlation with tumor tumor stage and histological grade. **E** The association of APCDDL1-AS1 expression with the RFS of these 54 ccRCC patients. **F** Comparison of APCDD1L-AS1 expression in the control HK2 cell line and six RCC cell lines (A498, ACHN, OSRC2, Caki-1, 786-O and RCC4). ^*^P< 0.05, ^**^P< 0.01.

First, we analyzed APCDD1L-AS1 expression in ccRCC and AN tissue using the transcriptome sequencing data of TCGA-KIRC, and the paired and unpaired t test results indicated that APCDD1L-AS1 expression was significantly reduced in ccRCC compared with AN tissue (**Fig. 1A**). Besides, we also compared the expression of APCDD1L-AS1 in ccRCC and AN tissue using the transcriptome sequencing data of two GEO DataSets (GSE53757 and GSE66272) and obtained the consistent results (**Fig. 1B**). Moreover, we used qRT-PCR to examine the expression of APCDD1L-AS1 in 54 pairs of ccRCC and AN tissue, and the analysis results identified the low expression status of APCDD1L-AS1 in ccRCC (**Fig. 1C**). Combining the clinicopathological and survival information of these 54 patients, our analysis results showed that the expression of APCDD1L-AS1 was lower in ccRCC with higher tumor stage and histological grade (**Fig. 1D**) and patients in APCDD1L-AS1 low expression group had a shorter RFS (Relapse-free survival) (**Fig. 1E**). The detailed clinical data of these 54 ccRCC patients are shown in **Table 1**. Finally, we examined the expression of APCDD1L-AS1 in the control HK2 cell line and six RCC cell lines (A498, ACHN, OSRC2, Caki-1, 786-O and RCC4). Our results identified that APCDD1L-AS1 expression was significantly decreased in these RCC cell lines compared with HK2 cell line, and the expression of APCDD1L-AS1 was also remarkably reduced in 786-O and RCC4 cell lines (with a* VHL* mutant) compared with Caki-1 cell line (*VHL* wild-type) (**Fig. 1F**).

### LncRNA APCDD1L-AS1 overexpression promoted cell apoptosis and weakened cell proliferation, migration and invasion *in vitro*

**Figure 2:**
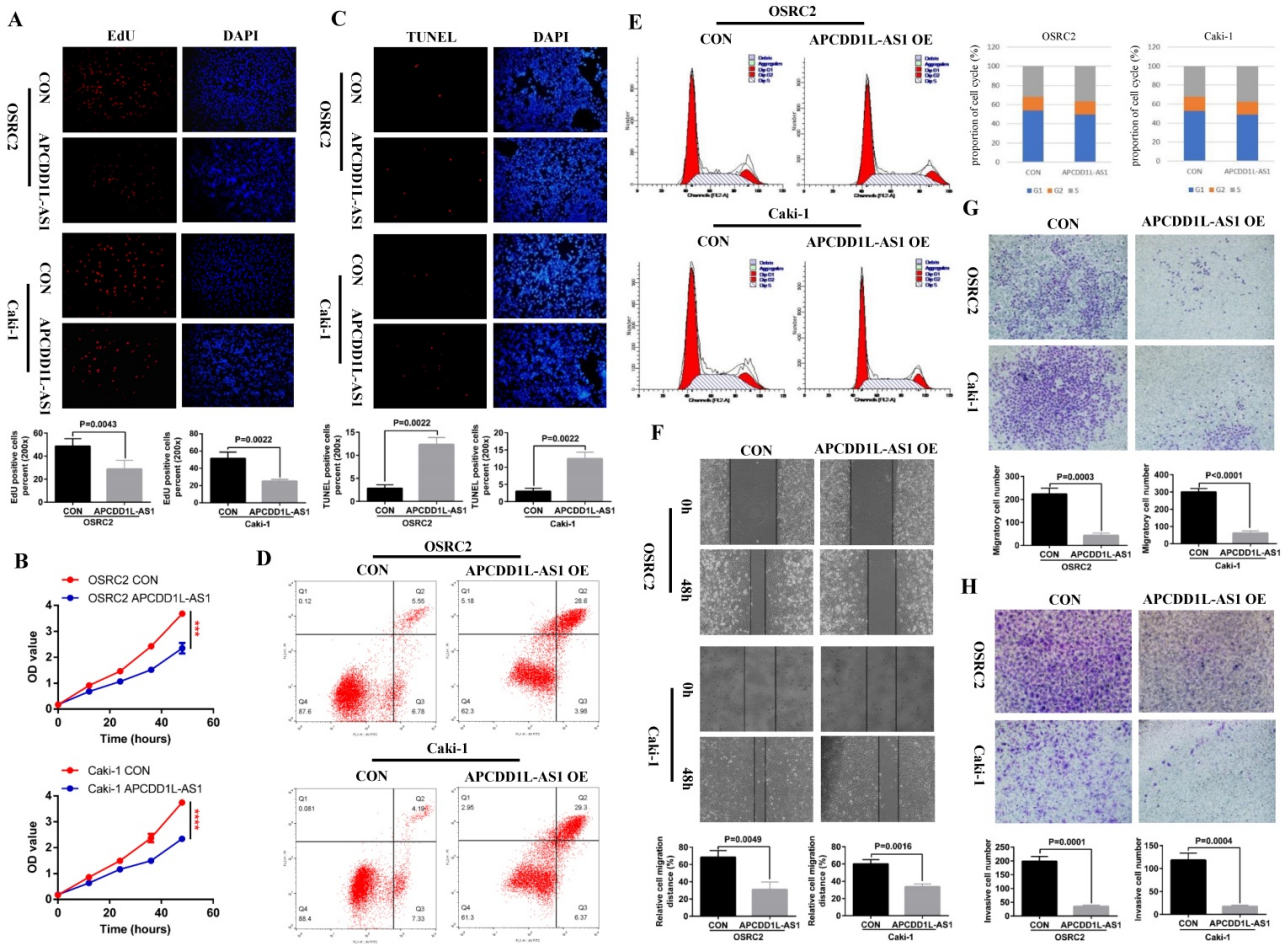
** APCDD1L-AS1 overexpression promoted cell apoptosis and weakened cell proliferation, migration and invasion *in vitro*. A** Comparison of the proportion of EdU positive cells in the APCDD1L-AS1 overexpression and the control OSRC2 and Caki-1 cells by immunofluorescence (200X). **B** Comparison of the proliferation ability of APCDD1L-AS1 overexpression and the control OSRC2 and Caki-1 cells by MTT assay. **C** Comparison of the proportion of TUNEL positive cells in the APCDD1L-AS1 overexpression and the control OSRC2 and Caki-1 cells by immunofluorescence (200X). **D** Comparison of the apoptosis of APCDD1L-AS1 overexpression and the control OSRC2 and Caki-1 cells by flow cytometry assay. **E** The proportion of cell cycle of APCDD1L-AS1 overexpression and the control OSRC2 and Caki-1 cells. **F** wound healing assay determined the migratory distances of APCDD1L-AS1 overexpressed OSRC2 and Caki-1 cells and their control cells (100X). **G and H** Comparison of cell migration and cell invasion ability in the APCDD1L-AS1 overexpression and the control Caki-1 and OSRC2 cells (100X). ^***^P< 0.001, ^****^P< 0.0001.

According to the results of the previous section, we have determined the low expression state of APCDD1L-AS1 in ccRCC, and then we needed to know whether overexpression of APCDD1L-AS1 affects the growth state of ccRCC cells? To this end, we constructed OSRC2 and Caki-1 cell lines stably transfected with APCDD1L-AS1. The effect of APCDD1L-AS1 expression increase on the proliferation of ccRCC cells was detected by immunofluorescence EdU method and MTT assay, and the effect of APCDD1L-AS1 expression increase on ccRCC cells apoptosis was detected by immunofluorescence TUNEL method and flow cytometry assay. The cell cycle was also detected. The effect of APCDD1L-AS1 expression increase on the invasion and metastasis ability of ccRCC cells was detected by cell wound healing, migration and invasion assays. Our results indicated that overexpression of APCDD1L-AS1 significantly restrained cell growth and increased cell apoptosis in OSRC2 and Caki-1 cells (**Fig. 2A-D**). The results of cell cycle assay showed that cells in S-phase were slightly increased in the APCDD1L-AS1 overexpression OSRC2 and Caki-1 cells compared with their control cells (**Fig. 2E**). Besides, APCDD1L-AS1 overexpression also attenuated the migration and invasion abilities of OSRC2 and Caki-1 cells (**Fig. 2F-H**).

### LncRNA APCDD1L-AS1 overexpression promoted cell apoptosis and attenuated cell proliferation and lung metastasis *in vivo*

**Figure 3:**
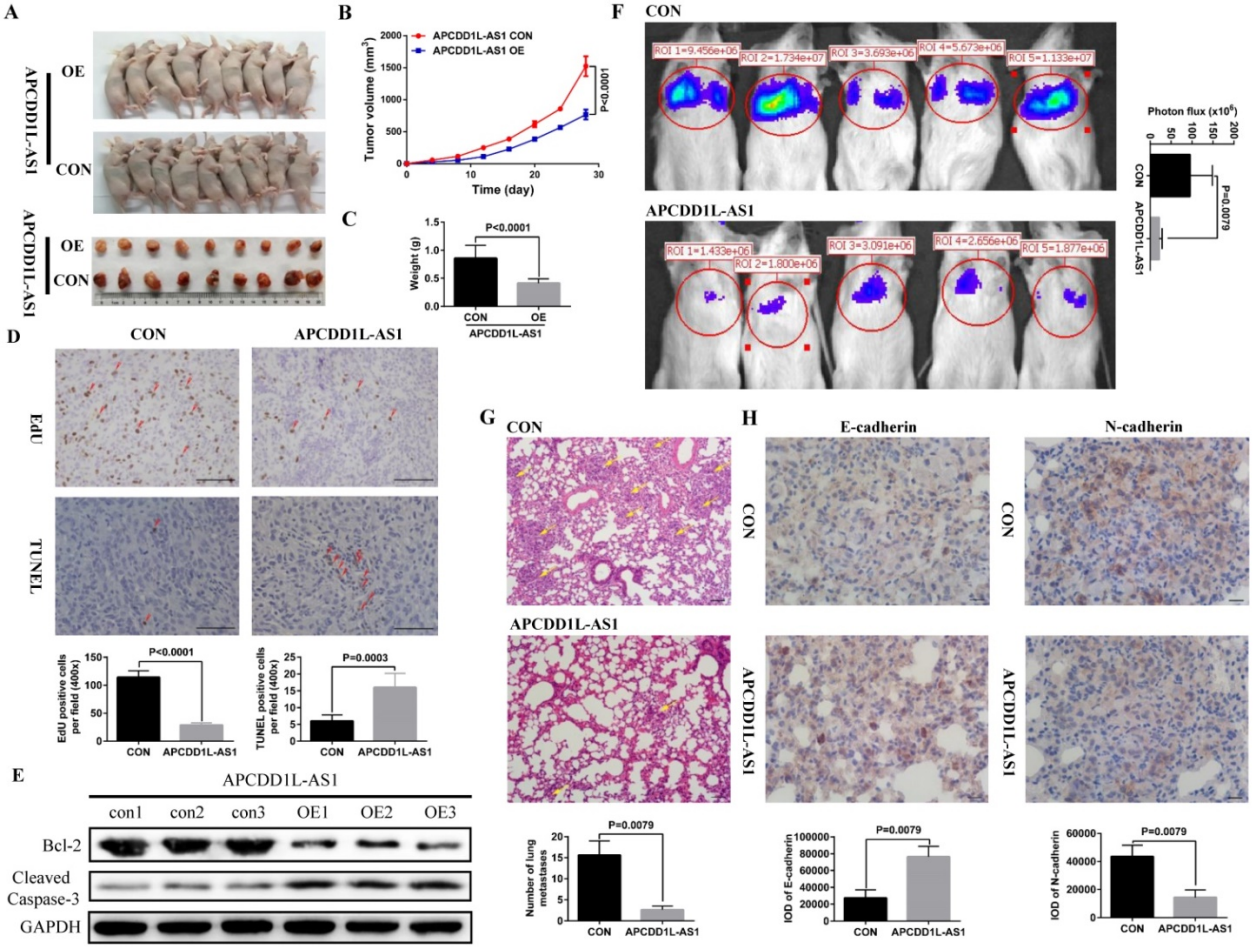
** APCDD1L-AS1 overexpression promoted cell apoptosis and attenuated cell proliferation and lung metastasis *in vivo*. A** Tumors collected from mice. **B and C** Tumor volume curves and tumor weights of the APCDD1L-AS1 overexpressed and the control groups were measured and compared. **D** The cell proliferation and apoptosis of tumors were examined. **E** The protein expression of Cleaved Caspase-3 was increased and Bcl-2 expression was decreased in the APCDD1L-AS1 overexpressed group. **F** The luciferase signals in the APCDD1L-AS1 overexpressed group were remarkably lower than those in the control group. **G** The results of hematoxylin-eosin staining of mice lung tissue. **H** APCDD1L-AS1 overexpression increased E-cadherin expression and decreased N-cadherin expression in mice pulmonary metastases.

To further verify the effect of increased APCDD1L-AS1 expression on the phenotype of ccRCC cells, we constructed ccRCC cells xenograft and lung metastasis mice models. Our xenograft experiment results showed that the tumor growth in the APCDD1L-AS1 overexpressed group was significantly slower than that in the control group (**Fig. 3A-C**), and the results of immunohistochemistry also indicated that cell proliferation was decreased and cell apoptosis was increased in APCDD1L-AS1 overexpressed tumors compared with the control tumors (**Fig. 3D**). Besides, compared with the control group, the protein expression of Cleaved Caspase-3 was increased and Bcl-2 expression was decreased in the APCDD1L-AS1 overexpressed group (Fig. 3E). The mice lung metastasis experiments results determined that the luciferase signal in the mouse lung was significantly attenuated after APCDD1L-AS1 overexpression (**Fig. 3F**), and the results of hematoxylin-eosin staining of lung tissue also identified that the number of lung metastases in mice was significantly reduced after APCDD1L-AS1 overexpression (**Fig. 3G**). Epithelial-mesenchymal transition (EMT) is a key factor in tumor progression, promoting tumor cell migration and invasion, thereby supporting metastasis 21. Thus, we also examined the expression of two EMT markers (E-cadherin and N-cadherin) in the lung metastases, and our immunohistochemistry results found that the expression of E-cadherin was increased and the expression of N-cadherin was decreased in the pulmonary metastases of APCDD1L-AS1 overexpressed group (**Fig. 3H**). Taken together, our results confirmed the role of APCDD1L-AS1 as a tumor suppressor gene in ccRCC.

### DNA methylation regulated the expression of lncRNA APCDD1L-AS1

**Figure 4:**
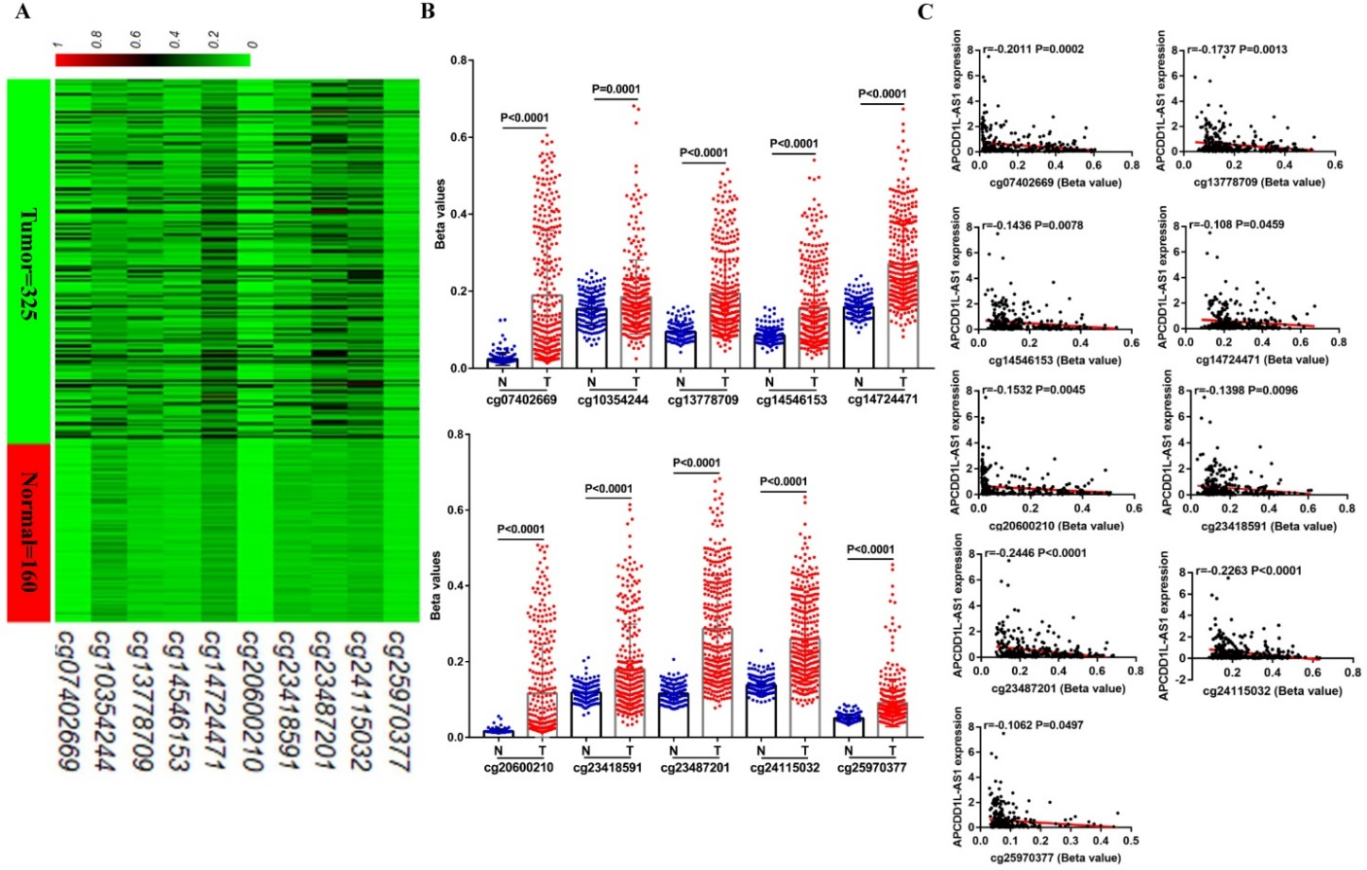
** DNA methylation level of APCDD1L-AS1 DNA was enhanced in ccRCC. A and B** Heatmap and statistical comparison of the difference in the DNA methylation levels of 10 CpG sites of APCDD1L-AS1 promoter in ccRCC (n=325) and AN tissue (n=160). **C** The correlation between the DNA methylation levels of these CpG sites and APCDD1L-AS1 expression.

Now that we have demonstrated the role of reduced APCDD1L-AS1 in ccRCC, what are the mechanisms that lead to the decrease of APCDD1L-AS1? DNA methylation, as an epigenetic modification, can include global hypomethylation and regional hypermethylation, in which regional hypermethylation is usually associated with gene silencing. Previous studies have also pointed out that the decreased expression of lncRNA SNHG3, SNHG15 and ZNF582-AS1 caused by DNA hypermethylation were closely related to the progression of ccRCC 15, 20. Thus, we obtained the methylation data of 10 CpG sites (cg07402669, cg10354244, cg13778709, cg14546153, cg14724471, cg20600210, cg23418591, cg23487201, cg24115032 and cg25970377) in APCDD1L-AS1 DNA from TCGA-KIRC. All of these CpG sites were located in the promoter region of APCDD1L-AS1, and the specific information of these CpG sites was shown in **Table 2**. Our analysis results suggested that the DNA methylation levels of all of these CpG sites were significantly higher in ccRCC compared with AN tissue (**Fig. 4A and B**), and the DNA methylation levels of nine of these 10 CpG sites were significantly negatively correlated with the APCDD1L-AS1 expression level (**Fig. 4C**).

**Figure 5:**
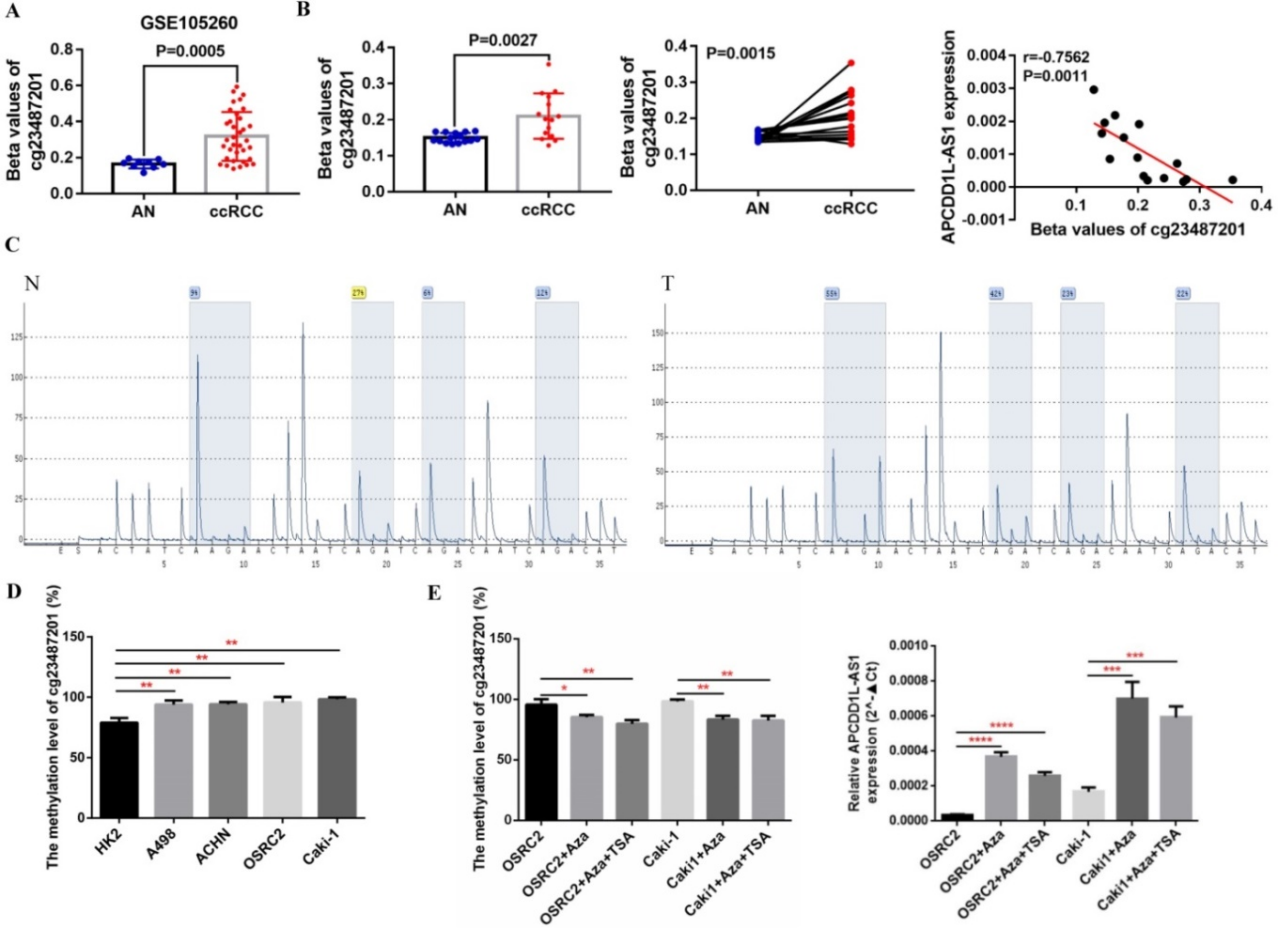
** APCDD1L-AS1 expression was negatively regulated by DNA hypermethylation. A** Comparison of the DNA methylation levels of cg23487201 in ccRCC (n=35) and AN (n=9) based on GSE105260 data. **B** Test of the DNA methylation levels of cg23487201 in 15 pairs of ccRCC and AN tissue through pyrosequencing. **C** Representative images of the pyrosequencing results. **D** Test of the DNA methylation levels of cg23487201 in HK2 cell lines and RCC cell lines. **E** Treatment with 5-aza-dC and TSA demethylated APCDD1L-AS1 promoter and increased APCDD1L-AS1 expression in OSRC2 and Caki-1 cells. N: adjacent normal renal tissue, T: ccRCC, ^*^P< 0.05,^ **^P < 0.01, ^***^P < 0.001, ^****^P < 0.0001.

Additionally, the DNA methylation level of cg23487201, whose DNA methylation level was most negatively correlated with APCDD1L-AS1 expression based on TCGA-KIRC data, was also higher in ccRCC compared with AN tissue based on the data from GSE105260 (**Fig. 5A**). Besides, we examined the methylation level of cg23487201 in 15 pairs of clinical ccRCC and AN tissue through pyrosequencing. The pyrosequencing results also determined that cg23487201 methylation level was significantly upregulated in ccRCC compared with AN tissue, and cg23487201 methylation level was significantly negatively correlated with APCDD1L-AS1 expression level (r=-0.7562, P=0.0011) (**Fig. 5B**). The representative results of pyrosequencing for cg23487201 in ccRCC and AN tissue were shown in **Fig. 5C**. In addition, the methylation level of cg23487201 in ccRCC cell lines were also tested, and our results identified that cg23487201 DNA methylation level was generally upregulated in ccRCC cells (**Fig. 5D**). Furthermore, the expression of APCDD1L-AS1 was remarkably increased after 5-Aza-2′-deoxycytidine (5-aza-dC, 5-Aza, A) and Trichostatin A (TSA, T) induced demethylation of APCDD1L-AS1 promoter in OSRC2 and Caki-1 cells (**Fig. 5E**). Thus, DNA hypermethylation may be one of the important causes for the downregulation of APCDD1L-AS1 expression in ccRCC.

### VHL protein affected the expression of lncRNA APCDD1L-AS1

**Figure 6:**
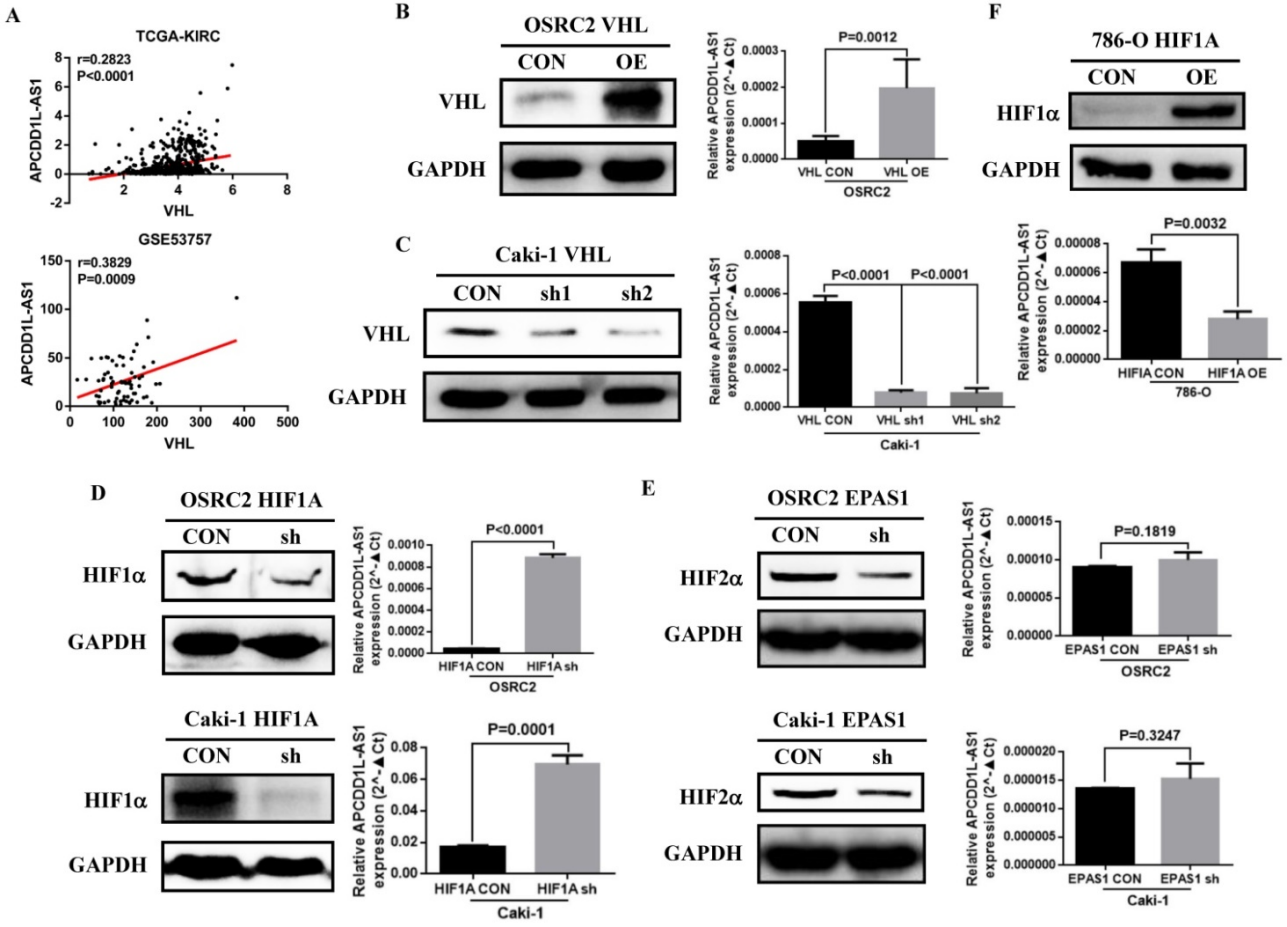
** VHL protein expression affected the expression of APCDD1L-AS1. A** The correlation between VHL expression and APCDD1L-AS1 expression based on TCGA-KIRC and GSE53757 data. **B** Comparison of APCDD1L-AS1 expression in VHL overexpressed OSRC2 cells and its control cells. **C** Comparison of APCDD1L-AS1 expression in VHL knockdown Caki-1 cells and its control cells. **D** Comparison of APCDD1L-AS1 expression in HIF1α knockdown OSRC2 and Caki-1 cells and their control cells. **E** Comparison of APCDD1L-AS1 expression in HIF2α knockdown OSRC2 and Caki-1 cells and their control cells. **F** Comparison of APCDD1L-AS1 expression in HIF1α overexpression 786-O cells and its control cells.

*VHL* gene inactivation is known to be by far the most common oncogenic driver event in ccRCC 22. Previous studies also indicated that the expression of many lncRNAs that play important roles in renal cancer were regulated by VHL or HIFs 23. By analyzing the RNA-seq data from TCGA-KIRC and GSE53757, we found that the expression of APCDD1L-AS1 was significantly positively correlated with the expression of VHL (**Fig. 6A**). To explore the specific relationship between VHL and APCDD1L-AS1, we overexpressed VHL expression in OSRC2 cells and knocked down VHL expression in Caki-1 cells. The qRT-PCR results showed that APCDD1L-AS1 expression was significantly increased after overexpression of VHL in OSRC2 cells (**Fig. 6B**), while APCDD1L-AS1 expression was significantly decreased after knockdown of VHL expression in Caki-1 cells (**Fig. 6C**). To further explore whether VHL protein affects the expression of APCDD1L-AS1 by regulating HIFs, we knocked down the expression of HIF1α and HIF2α in OSRC2 and Caki-1 cells. The qRT-PCR results demonstrated that APCDD1L-AS1 expression was significantly increased after knockdown of HIF1α expression in OSRC2 and Caki-1 cells (**Fig. 6D**), whereas there was no obvious alteration in APCDD1L-AS1 expression after knockdown of HIF2α expression in OSRC2 and Caki-1 cells (**Fig. 6E**). Furthermore, after overexpression of HIF1α in 786-O cells, APCDD1L-AS1 expression was significantly downregulated (**Fig. 6F**). Therefore, the above results determined that APCDD1L-AS1 expression could be regulated by VHL/HIF1α axis in ccRCC.

### APCDD1L-AS1 overexpression induced histones expression disorders

**Figure 7:**
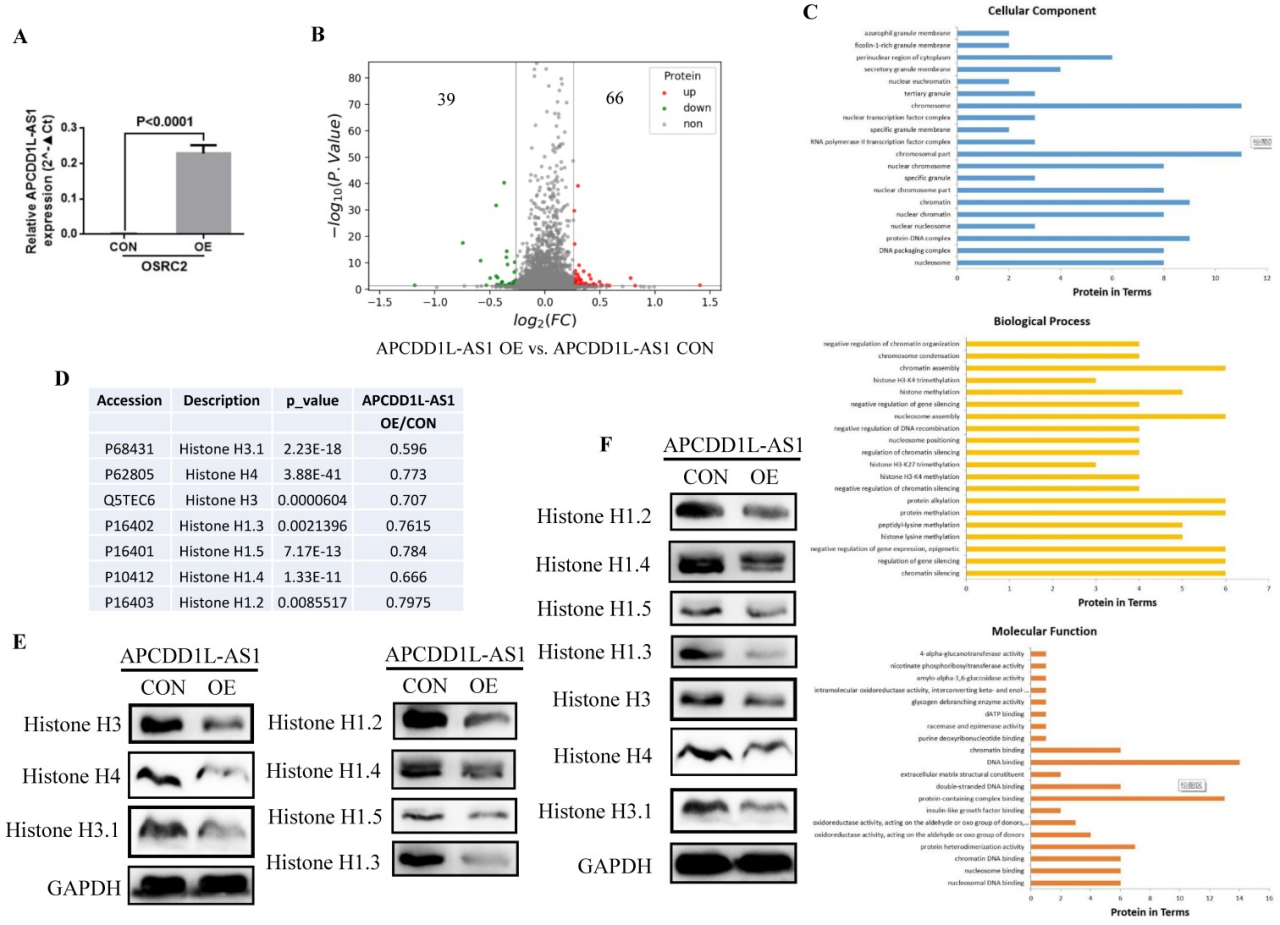
** APCDD1L-AS1 overexpression induced histones expression disorders. A The overexpression efficiency of APCDD1L-AS1 in OSRC2 cells. B** The TMT results showed that 39 proteins were downregulated and 66 proteins were upregulated in APCDD1L-AS1 overexpressed OSRC2 cells compared with its control cells. **C** Biological Process GO term enrichment analysis results of the complete 105 statistically significant proteins. **D** The TMT results of Histone H3.1, Histone H4, Histone H3, Histone H1.3, Histone H1.5, Histone H1.4 and Histone H1.2. **E** The protein expression of Histone H3.1, Histone H4, Histone H3, Histone H1.3, Histone H1.5, Histone H1.4 and Histone H1.2 in the same cell protein samples. **F** The protein expression of Histone H3.1, Histone H4, Histone H3, Histone H1.3, Histone H1.5, Histone H1.4 and Histone H1.2 in the in mice tumors.

Then, we utilized the TMT method to explore the potential downstream targets of APCDD1L-AS1 and its mechanism of action. ANOVA variance was used to evaluate the significance of differences in the TMT results, and the proteins with p value less than 0.05, ratio≥1.2 or ratio≤0.83 were regarded as differential proteins. The analysis results indicated a total of 105 differential proteins, including 66 upregulated proteins and 39 downregulated proteins in APCDD1L-AS1 overexpressed cells (**Fig. 7A and B**). Besides, the Biological Process GO term enrichment analysis of these 105 statistically significant proteins revealed that P68431 (Histone H3.1), P62805 (Histone H4), Q5TEC6 (Histone H3), P16402 (Histone H1.3), P16401 (Histone H1.5), P10412 (Histone H1.4), P16403 (Histone H1.2) proteins were included in the most of the top 20 enriched terms of Cellular Component, Biological Process and Molecular Function (**Fig. 7C and Table 3**). Moreover, we used western blot to examine the expression of these six proteins (Histone H3.1, Histone H4, Histone H3, Histone H1.3, Histone H1.5, Histone H1.4 and Histone H1.2) in the same cell protein samples. Consistent with the results of TMT (**Fig. 7D**), the western blot results identified that the expression of these six histones was remarkably lower in APCDD1L-AS1 overexpressed cells (**Fig. 7E**). Furthermore, we also examined the expression of these six histones in mice tumors by western blot and obtained the same results (**Fig. 7F**).

## Discussion

Several recent studies have shown that lncRNAs can function as tumor suppressor genes in ccRCC. For instance, ADAMTS9-AS2 inhibited cell proliferation and decreased chemoresistance in ccRCC 24; lnc-DILC stabilized PTEN and inhibited the progression of ccRCC 25; lncRNA MAGI2-AS3 restrained tumor progression and angiogenesis by regulating ACY1 in ccRCC 26. In the study, we identified a novel lncRNA, APCDD1L-AS1, which was downregulated in ccRCC, and overexpression of APCDD1L-AS1 restrained the growth and metastasis of ccRCC cells. However, previous studies have pointed out that APCDD1L-AS1 may also function as an oncogene in other tumors. For example, APCDD1L-AS1 induced icotinib resistance in LUAD and resulted in 5-fluorouracil resistance in OSCC 18, 19. Thus, APCDD1L-AS1 may play different roles in different tumors.

DNA methylation is a key epigenetic regulator of gene expression and well known as impactful makers for cancer detection 27. It is well known that epigenetic alterations, such as DNA methylation of promoter, can play a crucial role in renal tumorigenesis by silencing tumor suppressor genes 28. In RCC, promoters of >200 genes have been reported to be methylated, and many of these frequently methylated genes in RCC influence cancer hallmarks, including invasion, cell cycle regulation, apoptosis, and cell metabolism 29. In the present study, we found the DNA methylation levels of 10 CpG sites in APCDD1L-AS1 promoter were significantly upregulated in ccRCC, and the DNA methylation levels of nine of these 10 CpG sites were significantly negatively correlated with the expression levels of APCDD1L-AS1. Moreover, APCDD1L-AS1 expression in ccRCC cells was significantly increased after demethylation of APCDD1L-AS1 promoter.

Recently, there is increasing evidence that hypoxia promotes tumor progression and resistance to treatment, including ccRCC, pheochromocytoma and paraganglioma 30. Besides, hypoxia is also one of the major drivers of metabolic changes in ischemic disease and myocardial infarction 31. It can be seen that hypoxia not only plays an important role in tumors, but also affects the progression of non-tumor diseases. Therefore, it is very important to explore new hypoxia sensing pathway. Loss of VHL protein function leads to the activation of HIFs pathway, thereby activating hundreds of genes involved in many oncogenic pathways, which is one of the important mechanisms to promote the development or progression of ccRCC 32. Previous studies have pointed out that several lncRNAs that play vital roles in renal cancer are regulated by the VHL/HIFs axis. SARCC could inhibit the progression of hypoxic cell cycle in the VHL-mutant RCC cells while derepress it in the VHL-restored RCC cells 33. NICI was highly expressed in ccRCC, and its expression could be regulated by VHL 34. PVT1 interacted with HIF2α protein to enhance its stability by protecting it from ubiquitin-dependent degradation, thereby contributing to the development and metastasis of ccRCC 16. During this study, our result determined that overexpression of VHL upregulated the expression of APCDDL-AS1 and knockdown of VHL inhibited APCDD1L-AS1 expression. Moreover, APCDD1L-AS1 expression was increased after knockdown of HIF1α and decreased after overexpression of HIF1α. Similarly, previous study has indicated that lncRNA SNHG11 expression was also regulated by DNA methylation in colorectal cancer and could interact with and stabilize HIF1α and upregulate the expression of HIF1α target genes 35. Hence, our results confirmed that APCDD1L-AS1 was a downstream target regulated by both DNA methylation and the VHL/HIF1α axis in ccRCC.

Although our results suggested that APCDD1L-AS1 expression was significantly increased after knockdown of HIF1α expression, the mechanism by which HIF1α regulates APCDD1L-AS1 remains unclear. Previous studies have indicated that HIF1α could transcriptionally activate the expression of lncRNA. For instance, HIF1α directly bond to the promoter of KDM4A-AS1 and upregulated it expression in hepatocellular carcinoma 36, and HIF1α upregulated PVT1 transcription by binding to its promoter region in pancreatic cancer 37. Whether HIF1α can transcriptionally regulate the expression of APCDD1L-AS by binding to its promoter will be addressed in the near future.

Chromatin, a complex of DNA polymers and associated proteins, is the physiological substrate for all genomic processes, including gene expression, damage repair, replication, and the organization and segregation of chromosomes. The major components of chromatin are histones, including four core isoforms, H2A, H2B, H3, and H4, which together with associated DNA make up nucleosomes, and H1-linked histones, which associate with nucleosomes in dyads Combine at or near the shaft to form “chromosome” particle 38, 39. Previous several studies have also suggested that histone alterations are important causes of specific cancers, including Histone H3 and H3.3 alterations in glioblastoma 40, Histone H3.1 change in a congenital-onset soft tissue neoplasm 41, and histone H1 mutation in lymphoma 42. Besides, histone modifications seem to play an important role in renal cancer progression and can serve as prognostic biomarkers. Total histone H4 acetylation level was inversely correlated with pathological stage and nuclear grade, and decreased H3 acetylation level was correlated with systemic metastatic spread and tumor progression in RCC 43; lower total acetylation level of lysine 18 of histone H3 was associated with poorer survival in localized RCC 44. In addition, hypoxia could induce alterations in histone marks, which were associated with both repression and activation of genes 45. Previous study has also shown that hypoxia increased global demethylation level of lysine 9 of histone H3, thereby enhancing methyltransferase G9a activity, leading to gene repression in human embryonic renal cell line HEK293 46. In this study, the results of TMT and western blot confirmed that the expression of Histone H3.1, Histone H4, Histone H3, Histone H1.3, Histone H1.5, Histone H1.4 and Histone H1.2 were significantly reduced after APCDD1L-AS1 overexpression. Therefore, APCDD1L-AS1 may inhibit the growth and metastasis of ccRCC by causing the dysregulation of histones. Additionally, the mechanism of how APCDD1L-AS1 regulates the expression of histones will be more thoroughly investigated in the near future.

## Conclusions

Our study demonstrated that lncRNA APCDD1L-AS1 functioned as a tumor suppressor gene and its expression was downregulated in ccRCC. DNA hypermethylation and loss of VHL expression could lead to the decrease in APCDD1L-AS1 expression. APCDD1L-AS1 overexpression caused dysregulation of the expression of histones and restrained the development of ccRCC. Therefore, APCDD1L-AS1 may be a potential therapeutic target in ccRCC treatment.

## Figures and Tables

**Table 1:** The clinicopathologic characteristics of 54 ccRCC patients

Clinicopathologic characteristics	n (%)
**Age**	
<60	29 (53.7)
≥60	25 (46.3)
**Tumor size**	
< 2cm	7 (13.0)
≥ 2cm, < 5cm	29 (53.7)
≥ 5cm	18 (33.3)
**Gender**	
Male	39 (72.2)
Female	15 (27.8)
**Tumor stage**	
T1/T2	32 (59.3)
T3/T4	22 (40.7)
**Histological grade**	
G1/G2	36 (66.7)
G3/G4	18 (33.3)
**Relapse-free survival**	
Non-replased	41 (75.9)
Replased	13 (24.1)

**Table 2:** The detailed information of 10 CpG sites in APCDD1L-AS1 DNA based on TCGA-KIRC data

Composite Element REF	Chromosome	Start	End	CGI_Coordinate	Feature_Type
cg07402669	chr20	58514959	58514960	CGI:chr20:58514404-58515181	Island
cg10354244	chr20	58515034	58515035	CGI:chr20:58514404-58515181	Island
cg13778709	chr20	58514905	58514906	CGI:chr20:58514404-58515181	Island
cg14546153	chr20	58515259	58515260	CGI:chr20:58514404-58515181	S_Shore
cg14724471	chr20	58514405	58514406	CGI:chr20:58514404-58515181	Island
cg20600210	chr20	58514957	58514958	CGI:chr20:58514404-58515181	Island
cg23418591	chr20	58515261	58515262	CGI:chr20:58514404-58515181	S_Shore
cg23487201	chr20	58515066	58515067	CGI:chr20:58514404-58515181	Island
cg24115032	chr20	58514897	58514898	CGI:chr20:58514404-58515181	Island
cg25970377	chr20	58514877	58514878	CGI:chr20:58514404-58515181	Island

**Table 3:** GO term enrichment analysis of the complete 105 statistically significant proteins

Term	Desc	p-value	Protein involve
**Cellular Component**			
GO:0000786	nucleosome	5.42657E-09	P68431, P62805, Q5TEC6, P16402
			P16401, P10412, P16403, Q8IUE6
GO:0044815	DNA packaging complex	1.94097E-08	P68431, P62805, Q5TEC6, P16402
			P16401, P10412, P16403, Q8IUE6
GO:0000788	nuclear nucleosome	3.42286E-05	P68431, P62805, P10412
GO:0000790	nuclear chromatin	0.000563299	P68431, P62805, P16402, P16401
			P10412, P16403, O00268, Q8IUE6,
GO:0000785	chromatin	0.0015933	P68431, P62805, Q5TEC6, P16402, P16401
			P10412, P16403, O00268, Q8IUE6
GO:0044454	nuclear chromosome part	0.002548084	P68431, P62805, P16402, P16401
			P10412, P16403, O00268, Q8IUE6
GO:0042581	specific granule	0.003470143	Q9NX76, Q9NQR4, Q5T4S7
GO:0000228	nuclear chromosome	0.004498937	P68431, P62805, P16402, P16401
			P10412, P16403, O00268, Q8IUE6
GO:0044427	chromosomal part	0.006060522	P68431, P62805, Q5TEC6, P16402
			P16401, P10412, B7Z7S9, P16403
			O00268, Q8IUE6, Q9NVF7
GO:0090575	RNA polymerase II	0.01167711	O00268, P25208, A0A0B4J1Z5
	transcription factor complex		
GO:0035579	specific granule membrane	0.01464028	Q9NX76, Q5T4S7
GO:0044798	nuclear transcription factor complex	0.01664205	O00268, P25208, A0A0B4J1Z5
GO:0005694	chromosome	0.01855285	P68431, P62805, Q5TEC6, P16402
			P16401, P10412, B7Z7S9, P16403
			O00268, Q8IUE6, Q9NVF7
GO:0070820	tertiary granule	0.01950291	Q9NQR4, Q5T9A4, Q5T4S7
GO:0005719	nuclear euchromatin	0.02006129	P16402, P16403
GO:0030667	secretory granule membrane	0.02145841	Q9NX76, O95716, Q5T9A4, Q5T4S7
GO:0048471	perinuclear region of cytoplasm	0.02266291	P04732, P16989, Q96J84, P15531
			Q9BR76, P15121
GO:0101003	ficolin-1-rich granule membrane	0.02618205	Q5T9A4, Q5T4S7
GO:0035577	azurophil granule membrane	0.02618205	Q9NX76, O95716
GO:0030659	cytoplasmic vesicle membrane	0.027415	Q9NX76, O75396, O95716
			Q5T9A4, Q5T4S7, G3V5X8
**Biological Process**			
GO:0006342	chromatin silencing	1.53002E-06	P68431, P62805, P16402
			P16401, P10412, P16403
GO:0060968	regulation of gene silencing	4.22941E-06	P68431, P62805, P16402
			P16401, P10412, P16403
GO:0045814	negative regulation of gene expression, epigenetic	4.22941E-06	P68431, P62805, P16402
			P16401, P10412, P16403
GO:0034968	histone lysine methylation	1.35777E-05	P16402, P16401, P10412
			P16403, Q9C005
GO:0018022	peptidyl-lysine methylation	1.35777E-05	P16402, P16401, P10412
			P16403, Q9C005
GO:0006479	protein methylation	1.4475E-05	P16402, P16401, P10412
			P16403, O95716, Q9C005
GO:0008213	protein alkylation	1.4475E-05	P16402, P16401, P10412
			P16403, O95716, Q9C005
GO:0031936	negative regulation of chromatin silencing	1.5545E-05	P16402, P16401, P10412, P16403
GO:0051568	histone H3-K4 methylation	1.5545E-05	P16402, P10412, P16403, Q9C005
GO:0098532	histone H3-K27 trimethylation	3.42286E-05	P16402, P10412, P16403
GO:0031935	regulation of chromatin silencing	3.53573E-05	P16402, P16401, P10412, P16403
GO:0016584	nucleosome positioning	3.53573E-05	P16402, P16401, P10412, P16403
GO:0045910	negative regulation of DNA recombination	3.53573E-05	P16402, P16401, P10412, P16403
GO:0006334	nucleosome assembly	5.22695E-05	P68431, P62805, P16402
			P16401, P10412, P16403
GO:0060969	negative regulation of gene silencing	6.89335E-05	P16402, P16401, P10412, P16403
GO:0016571	histone methylation	7.94447E-05	P16402, P16401, P10412
			P16403, Q9C005
GO:0080182	histone H3-K4 trimethylation	0.000133642	P16402, P10412, P16403
GO:0031497	chromatin assembly	0.000181423	P68431, P62805, P16402
			P16401, P10412, P16403
GO:0030261	chromosome condensation	0.000196527	P16402, P16401, P10412, P16403
GO:1905268	negative regulation of chromatin organization	0.000196527	P16402, P16401, P10412, P16403
**Molecular Function**			
GO:0031492	nucleosomal DNA binding	2.60664E-06	P68431, Q5TEC6, P16402
			P16401, P10412, P16403
GO:0031491	nucleosome binding	2.05966E-05	P68431, Q5TEC6, P16402
			P16401, P10412, P16403
GO:0031490	chromatin DNA binding	2.05966E-05	P68431, Q5TEC6, P16402
			P16401, P10412, P16403
GO:0046982	protein heterodimerization activity	0.00029803	P68431, P62805, Q5TEC6, O00268
			P25208, P51116, Q8IUE6
GO:0016903	oxidoreductase activity, acting on the aldehyde or oxo group of donors	0.000846244	P08559, A0A1B0GTY9
			Q5ZEY3, P15121
		
GO:0016620	oxidoreductase activity, acting on the aldehyde or oxo group of donors, NAD or NADP as acceptor	0.003470143	P08559, A0A1B0GTY9, Q5ZEY3
		
		
GO:0005520	insulin-like growth factor binding	0.006113907	Q16270, B3KRV6
GO:0044877	protein-containing complex binding	0.006324178	P68431, Q5TEC6, P16402, P16401, P10412
			P16403, Q13751, P16989, P25208
			P15531, P05386, Q9BR76, F8WBH5
GO:0003690	double-stranded DNA binding	0.007727656	P16402, P16401, P10412
			P16403, P16989, P15531
GO:0005201	extracellular matrix structural constituent	0.01464028	Q16270, Q13751
GO:0003677	DNA binding	0.01591233	P68431, P62805, Q5TEC6, P16402
			P16401, P10412, P16403, Q6DD87
			P16989, O00268, P25208, P15531
			O00287, Q8IUE6
GO:0003682	chromatin binding	0.01982527	P68431, Q5TEC6, P16402
			P16401, P10412, P16403
GO:0032554	purine deoxyribonucleotide binding	0.03277154	P10412
GO:0016854	racemase and epimerase activity	0.03277154	C9J6A7
GO:0032564	dATP binding	0.03277154	P10412
GO:0004133	glycogen debranching enzyme activity	0.03277154	A0A0M4G3H8
GO:0016862	intramolecular oxidoreductase activity interconverting keto- and enol-groups	0.03277154	P14174
		
GO:0004135	amylo-alpha-1,6-glucosidase activity	0.03277154	A0A0M4G3H8
GO:0004516	nicotinate phosphoribosyltransferase activity	0.03277154	C9J8U2
GO:0004134	4-alpha-glucanotransferase activity	0.03277154	A0A0M4G3H8

## Abbreviations

ccRCC: clear cell renal cell carcinoma
lncRNAs: long non-coding RNAs
VHL: von Hippel Lindau
HIFs: hypoxia-inducible factors
OSCC: oral squamous cell carcinoma
LUAD: lung adenocarcinoma
AN: adjacent normal renal
TCGA-KIRC: The Cancer Genome Atlas-Kidney Renal Clear Cell Carcinoma
GEO: Gene Expression Omnibus
qRT-PCR: Quantitative real-time PCR
TMT: Tandem mass tags
RFS: Relapse-free survival
EMT: Epithelial-mesenchymal transition
